# Awareness on oral health status of Indian women during pregnancy by gynaecologists

**DOI:** 10.6026/9732063002001804

**Published:** 2024-12-31

**Authors:** Manali Dande, Mahima Tyagi, Anupama Arya, Karuna Meravi, Manisha Mohanty, Sushil Kumar Sahoo

**Affiliations:** 1 Obstetrician and Gynecologist, Medcare Women and Children, Hospital, Dubai, UAE; 2 Department of Oral Medicine and Radiology, DJ Dental College, Modinagar, Uttar Pradesh, India; 3 Department of Community Medicine, Government Doon Medical College, Dehradun, India; 4 Department of Obstetrics and Gynaecology, Sunderlal Patwa Government, Medical College, Mandsaur, Madhya Pradesh, India; 5 Private Consultant, Bhubaneswar, Odisha, India; 6 Department of Oral and Maxillofacial Surgery, Hi-Tech Dental College and Hospital, Bhubaneswar, Odisha, India

**Keywords:** Oral health, pregnancy, gynecologists, periodontal disease, pregnancy gingivitis

## Abstract

The awareness of gynecologists on oral health conditions during pregnancy is of interest. Gynecologists generally acknowledge the importance of oral health during pregnancy. However, there is a need for improved awareness regarding specific conditions, guidelines the importance of routine oral health assessment. Educational interventions and collaborative efforts between gynecologists and dental professionals are crucial to ensure optimal oral health care for pregnant women.

## Background:

The complex physiological changes during pregnancy significantly impact oral health, necessitating increased attention from healthcare providers. Hormonal fluctuations, particularly elevated levels of estrogen and progesterone, can exacerbate existing oral conditions and increase susceptibility to new ones [1, 2]. These hormonal shifts contribute to heightened gingival inflammation, increased blood flow to the oral tissues altered immune responses, making pregnant women more prone to dental issues. Periodontal disease, a prevalent oral health concern during pregnancy, has been associated with adverse pregnancy outcomes. Research has linked this inflammatory condition to an increased risk of preterm birth, low birth weight infants preeclampsia [3, 4, 5-6]. The potential mechanisms behind these associations include the systemic spread of oral pathogens and inflammatory mediators, which may impact fetal development and placental function. Pregnancy gingivitis, characterised by gingival inflammation and bleeding, affects a significant proportion of expectant mothers [7]. This condition, if left untreated, can progress to more severe periodontal disease. The increased prevalence of oral health issues during pregnancy underscores the importance of regular dental check-ups and good oral hygiene practices throughout gestation. Gynecologists are crucial in addressing oral health concerns during pregnancy [8]. As primary care providers for expectant mothers, they are uniquely positioned to integrate oral health screenings, education referrals into routine prenatal care. Early identification and management of oral health issues can significantly improve maternal and fetal outcomes [9]. Studies have demonstrated that incorporating oral health education and dental referrals into prenatal care programs can improve oral health outcomes for mothers and their children [10, 11]. Despite the growing body of evidence highlighting the importance of oral health during pregnancy, there remains a knowledge gap among healthcare providers, including gynecologists [12, 13-14]. This lack of awareness can result in missed opportunities for early intervention, patient education preventive care [15]. Therefore, it is of interest to report the awareness of gynecologists on oral health conditions during pregnancy.

## Methods and Materials:

## Study design and setting:

This cross-sectional study was conducted among gynecologists practicing in various hospitals and clinics.

## Study population and sampling:

The study population included all registered gynecologists actively practicing within the defined geographical area, regardless of their specialization within the field. Due to logistical constraints in accessing the complete list of eligible gynecologists, a convenience sampling method was employed. During non-patient care hours, gynaecologists were approached in person at their workplaces (hospitals and clinics). The study objectives and procedures were explained in detail those who expressed interest and met the inclusion criteria were invited to participate. Participants provided written informed consent before proceeding with the questionnaire.

## Inclusion criteria:

[1] Registered gynaecologist actively practicing within the defined geographical area.

[2] Willingness to participate and provide informed consent.

## Exclusion criteria:

[1] Gynecologists unavailable during the study period (*e.g.*, on leave, sabbatical).

[2] Gynecologists who declined to participate.

## Data collection instrument:

A structured, self-administered questionnaire was developed to collect data for this study. The questionnaire was meticulously crafted based on a comprehensive review of existing literature on oral health in pregnancy and consultation with experts in both gynaecology and dentistry. This ensured the questionnaire's content validity and relevance to the study objectives. Before its use in the main study, the questionnaire underwent rigorous pre-testing. A pilot study was conducted with a small group of gynecologists (n=10) who were not part of the main study sample. The pilot study participants were asked to complete the questionnaire and provide feedback on its clarity, comprehensiveness ease of understanding. Their feedback was carefully analysed necessary revisions were made to the questionnaire to enhance its clarity, flow overall quality.

## The finalised questionnaire consisted of the following sections:

## Demographics:

This section collected basic demographic information about the participating gynecologists, including:

[1] Age (recorded in years)

[2] Gender (male/female)

[3] Years of experience as a practicing gynaecologist (recorded in years)

[4] Practice setting (categorised as public hospital, private hospital, or private clinic)

## Knowledge of oral health in pregnancy:

This section assessed the gynaecologists' knowledge and understanding of oral health issues commonly encountered during pregnancy. It included questions related to:

[1] Pregnancy gingivitis: Causes, symptoms, risk factors and potential impact on pregnancy outcomes.

[2] Periodontal disease: Types, causes, symptoms, risk factors potential impact on pregnancy outcomes.

[3] The influence of hormonal changes during pregnancy on oral health.

[4] The relationship between oral health and overall systemic health during pregnancy.

## Awareness of oral health guidelines:

This section evaluated the gynaecologists' familiarity with established national and international guidelines for managing oral health during pregnancy. Questions focused on:

[1] Awareness of specific oral health guidelines for pregnant women.

[2] Knowledge of recommended oral hygiene practices during pregnancy.

[3] Understanding of the role of dental referrals during pregnancy.

## Practices regarding oral health:

This section explored the gynaecologists' current practices and approaches to integrating oral health into routine care for pregnant patients. Questions addressed:

[1] Frequency of oral health screening during prenatal visits.

[2] Methods used for oral health assessment (*e.g.*, visual inspection, inquiries about symptoms).

[3] Provision of oral health counselling and education to pregnant patients.

[4] Referral practices to dental professionals for specialised care.

[5] Perceived barriers to providing comprehensive oral health care to pregnant women.

## Data analysis:

Once data collection was complete, all responses were carefully entered into a dedicated database created using SPSS 23. The data underwent thorough cleaning to identify and rectify any potential errors or inconsistencies in data entry. Descriptive statistics were employed to analyse the collected data. Frequency distributions and percentages were calculated for categorical variables, providing a clear picture of the distribution of responses within each category. For continuous variables, such as age and years of experience, means and standard deviations were calculated to describe the central tendency and variability of the data. Further statistical analyses, such as chi-square tests or t-tests, may be conducted to explore potential associations between demographic characteristics, knowledge levels reported practices. However, the specific statistical tests employed will depend on the nature of the data and the research questions being addressed.

## Results:

## Demographic characteristics of participants:

A total of 200 gynecologists participated in the study. The demographic characteristics are summarised in Table 1.

## Knowledge of oral health issues in pregnancy:

The knowledge of gynecologists regarding common oral health conditions during pregnancy, including pregnancy gingivitis and periodontal disease, is shown in Table 2.

## Awareness of oral health guidelines:

The awareness of oral health guidelines for pregnant women among gynecologists was evaluated, as summarised in Table 3.

## Practices regarding oral health screening and referrals:

The practices of gynecologists in integrating oral health into prenatal care are shown in Table 4. A significant association was observed between years of experience and knowledge of oral health issues during pregnancy. Gynecologists with over 10 years of experience demonstrated better knowledge of periodontal disease's impact on pregnancy outcomes (p < 0.05). However, there was no significant difference in knowledge scores between public and private practice settings (p > 0.05).The study identified significant knowledge gaps among gynecologists regarding oral health during pregnancy. While 70% were aware of pregnancy gingivitis, only 42.5% recognised the link between periodontal disease and adverse pregnancy outcomes like preterm birth. Awareness of oral health guidelines was also limited, with only 45% familiar with relevant national or international recommendations. Despite acknowledging the importance of oral health, only 35% routinely screened patients 40% provided education, indicating inconsistencies in practice. Barriers such as time constraints and unclear referral pathways were reported by 70% of participants, emphasising the need for better collaboration between medical and dental professionals to integrate oral health into prenatal care.

## Discussion:

The awareness and knowledge of gynecologists regarding oral health of Indian women during pregnancy is of interest. Our findings reveal a mixed picture, with a general awareness of the importance of oral health but significant gaps in specific knowledge and practices. Most gynecologists in our study acknowledged the potential impact of pregnancy on oral health. This finding aligns with previous studies reporting an increasing recognition of the oral-systemic link among healthcare providers [16, 17]. However, knowledge gaps were evident regarding specific oral health conditions and their management during pregnancy. For instance, a considerable proportion of gynecologists were unable to correctly identify the association between periodontal disease and adverse pregnancy outcomes, a well-established link supported by numerous studies [3, 4, 5-6]. This finding highlights the need for targeted education on the specific risks associated with periodontal disease during pregnancy, particularly its potential to contribute to preterm birth and low birth weight. Furthermore, a significant proportion of gynecologists were unfamiliar with recommended oral hygiene practices for pregnant women. This finding is concerning, as simple interventions like proper brushing, flossing techniques professional dental cleanings can significantly reduce the risk of oral health problems [9, 10]. It is crucial to educate gynecologists on these recommendations and empower them to provide practical advice to their patients. Our study also revealed a discrepancy between awareness and practice. While most gynecologists recognised the importance of oral health during pregnancy, a smaller proportion reported routinely screening their patients for oral health issues or providing referrals to dental professionals. This finding aligns with previous research indicating that oral health often receives inadequate attention during routine prenatal care [11, 12]. Several factors may contribute to this gap, including time constraints during consultations, lack of clear referral pathways perceived low priority of oral health compared to other pregnancy-related concerns [13, 14-15]. Addressing these barriers requires a multi-pronged approach. Integrating oral health education into gynaecology residency programs and continuing medical education courses can enhance knowledge and promote routine screening practices [16, 17]. Collaborative efforts between gynaecological and dental professional organisations can facilitate the development of clear referral pathways and shared care protocols [9]. Additionally, raising awareness among pregnant women about the importance of oral health and encouraging them to communicate any concerns to their healthcare providers is essential [10].

## Limitations:

A convenience sampling method may limit our findings' generalizability to the broader gynaecologist population. Future research utilising random sampling techniques would enhance the representativeness of the study sample. Additionally, self-reported data collected through questionnaires are susceptible to recall and social desirability biases. Objective assessments of knowledge and practices would provide a more accurate reflection of the current situation.

## Conclusion:

Data provides valuable insights into the current state of gynaecologists' awareness and knowledge regarding oral health during pregnancy. While there is a general understanding of the importance of oral health, significant gaps remain in specific knowledge areas and practices. Targeted educational interventions, inter professional collaboration patient empowerment are crucial to bridge these gaps and ensure optimal oral health care for pregnant women.

## Figures and Tables

**Table 1:** Demographic characteristics of gynecologists (n=200)

**Characteristic**	**Frequency (n)**	**Percentage (%)**
**Age**		
25-35 years	60	30
36-45 years	80	40
46-55 years	40	20
56 years and above	20	10
**Gender**		
Male	80	40
Female	120	60
**Years of Experience**		
Less than 5 years	40	20
5-10 years	80	40
More than 10 years	80	40
**Practice Setting**		
Public hospital	90	45
Private hospital	70	35
Private clinic	40	20

**Table 2:** Knowledge of oral health conditions during pregnancy (n=200)

**Knowledge Aspect**	**Correct Response (%)**	**Incorrect/Unaware (%)**
Pregnancy gingivitis: Symptoms and risks	140 (70.0%)	60 (30.0%)
Periodontal disease: Impact on pregnancy oral health	90 (45.0%)	110 (55.0%)
Hormonal changes influencing	130 (65.0%)	70 (35.0%)
The link between periodontal disease and preterm birth	85 (42.5%)	115 (57.5%)

**Table 3:** Awareness of oral health guidelines (n=200)

**Awareness Aspect**	**Aware (n, %)**	**Unaware (n, %)**
Knowledge of national/international guidelines	90 (45.0%)	110 (55.0%)
Awareness of recommended oral hygiene practices	120 (60.0%)	80 (40.0%)
Familiarity with dental referral practices	100 (50.0%)	100 (50.0%)

**Table 4:** Oral health screening and referral practices (n=200)

**Practice Aspect**	**Yes (n, %)**	**No (n, %)**
Routine oral health screening	70 (35.0%)	130 (65.0%)
Providing oral health education	80 (40.0%)	120 (60.0%)
Referral to dental professionals	90 (45.0%)	110 (55.0%)
Encountering barriers to oral health care	140 (70.0%)	60 (30.0%)
